# Simple Enzyme Immobilization for Flow Chemistry? An Assessment of Available Strategies for an Acetaldehyde-Dependent Aldolase

**DOI:** 10.3390/molecules27196483

**Published:** 2022-10-01

**Authors:** Martin Wäscher, Thomas Classen, Jörg Pietruszka

**Affiliations:** 1 Institute for Bioorganic Chemistry, Heinrich Heine University Düsseldorf, 40225 Düsseldorf, Germany; 2 Institute for Bio- and Geosciences 1: Bioorganic Chemistry, Forschungszentrum Jülich, 52425 Jülich, Germany

**Keywords:** aldolase, DERA, flow chemistry, enzyme stability, process optimization

## Abstract

Enzyme immobilization is a technology that enables (bio-)catalysts to be applied in continuous-flow systems. However, there is a plethora of immobilization methods available with individual advantages and disadvantages. Here, we assessed the influence of simple and readily available methods with respect to the performance of 2-deoxy-d-ribose-5-phosphate aldolase (DERA) in continuous-flow conditions. The investigated immobilization strategies cover the unspecific attachment to carriers via epoxides, affinity-based attachment via metal ion affinity, StrepTag™-StrepTactin™ interaction as well as the covalent affinity attachment of an enzyme to a matrix tethered by the HaloTag^®^. The metal-ion-affinity-based approach outperformed the other methods in terms of immobilized activity and stability under applied conditions. As most enzymes examined today already have a HisTag for purification purposes, effective immobilization may be applied, as simple as a standard purification, if needed.

## 1. Introduction

Flow chemistry is an emerging technology in organic chemistry with many advantages. For example, a reaction can be tightly controlled in terms of temperature, pressure and reaction time. Appropriate process design can compart reaction steps and therefore easily enable multistep reactions or simplify downstream processing, and compound library screenings can be conducted by automation [1]. Enzymes enrich the possibilities of a chemist further, often imparting high enantio-, diastereo-, chemo-, and regioselectivity. Additionally, reactions can be performed under mild conditions if needed [2]. Immobilized enzymes in packed bed reactors enable the combination of these advantages. In recent decades, many methods have been described to successfully immobilize enzymes [3,4,5].

The 2-deoxy-d-ribose-5-phosphate aldolase (DERA) catalyzes the retro-aldol cleavage of 2-deoxy-d-ribose-5-phosphate (DRP) towards acetaldehyde and glyceradehyde-3-phosphate in the deoxyribonucleic acid (DNA) degradation pathway [6,7]. It forms homodimers and is ubiquitous in nature. The DERA also works in the aldol direction. The double aldol adduct is mainly formed this way with two subsequent aldol additions of donor acetaldehyde to a substrate and intramolecular cyclization to the hemiacetal [8]. These products can be valuable intermediates for statin-side chain synthesis, for instance. In addition to the industrial relevance of the double aldol products, enantio-enriched products from single aldol reactions may be valuable intermediates, too. With a precise control of the contact time between enzyme and substrate, the mono-aldol product may be the predominant product, available for further in-line conversion to desired compounds. Hindges et al. successfully produced a β-hydroxy carboxylic acid, a 1,3-diol, a hydroxy homoallylalcohol and a hydroxylated secondary amine in an enantiopure or diastereomerically enhanced fashion, respectively, in a coupled flow/batch process. An α,β-unsaturated aldehyde in a flow cascade could also be obtained [9]. For this study, the *E. coli* DERA C47M A95C, modified for substrate tolerance and increased structural stability, was used. The C47M variation impairs covalent inhibition by crotonaldehyde—a side product formed from two acetaldehyde molecules [10]. The A95C variation strengthens the dimeric structure via an intermolecular disulfide bond [11].

The immobilization of DERA has been described as beneficial and successful in the past. Adsorption on mesoporous silica led to higher conversions by increased stability [12]. The covalent attachment of DERA on mesoporous silica via *p*-benzoquinone led to increased substrate and temperature resistance [13]. The additional modification of matrix protein conjugate via amino acids increased the relative activity [14]. Immobilized DERA on carbon nanotubes showed increased stability compared to free enzymes [15]. The immobilization on polymeric thin films retains the catalysts activity [16]. The hydrophobic self-assembly of DERA thin films was achieved by poly-isopropyl acrylamide conjugation [17,18]. Entrapment in an alginate matrix supported by a loofa sponge was investigated [19]. Even nanomagnetic particles are suitable for use in increasing substrate and temperature tolerance [20]. Entrapment in acrylic microgels is possible. However, a significant drop in activity was observed [21]. Nevertheless, a direct comparison of immobilization approaches for this enzyme could not be found.

**Figure 1:**
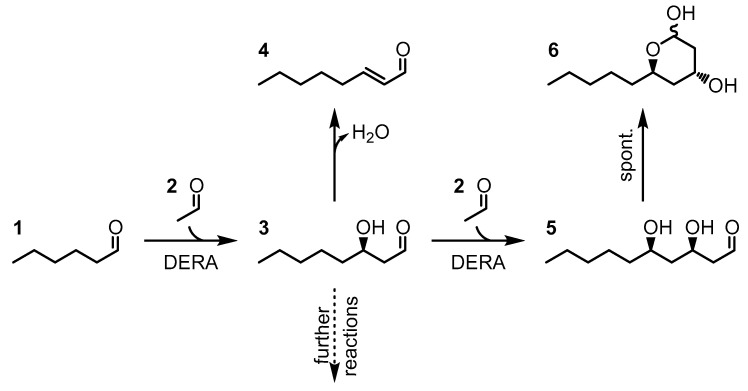
Reactions catalyzed via DERA, starting from hexanal (**1**). The DERA catalyzes the aldol addition between hexanal (**1**) and acetaldehyde (**2**), forming (*R*)-3-hydroxy octanal (**3**), which may be used in further reactions [9]. The aldol condensation results in oct-2-enal (**4**). *(R)*-3-hydroxy-octanal (**3**) can participate in a second aldol reaction, forming (*R*,*R*)-3,6-dihydroxy decanal (**5**), which spontaneously forms the hemiacetal 2,4-dideoxy-(*R*)-3-hydroxy-(*R*)-5-pentyl-pyranoside (**6**).

For this study, we focused on simple and practical approaches. Therefore, HaloTag^®^-based immobilization for the specific and covalent attachment of a catalyst onto a carrier via ester bonds was chosen as the standard [22,23,24]. As one-step purification and immobilization is a desired trait, non-covalent immobilization via the metal ion affinity (MIA) of His_6_-tag and His_6_Gly_2_Cys_2_-tag (HisNu-tag) and via StrepTag™-StrepTactin™ interaction were selected [25,26,27,28]. The epoxy acrylic resin *Immobead 150P* was chosen as an unselective matrix for covalent attachment [29]. To compare the immobilized DERA variants, packed bed reactors were prepared and used for the conversion of hexanal (**1**) and acetaldehyde (**2**) to 3-hydroxyoctanal (**3**). As fixed-reaction conditions were chosen for direct comparability, side products such as the aldol condensate oct-2-enal (**4**) and the cyclization products from 3,6-dihydroxy decanal (**5**) to 2,4-dideoxy-3(*R*)-hydroxy-5(*R*)-pentyl-pyranoside (**6**) are expected. (See the reaction scheme in Figure 1.)

## 2. Results

All observations were made with respect to the formation of (*R*)-3-hydroxy-octanal (**3**) from hexanal (**1**) and acetaldehyde (**2**) under fixed flow conditions. Therefore, the enzyme variants were immobilized as described in the Methods section, forming a ~350 µL packed bed reactor. The premixed reactants were then pumped through the reactor. The product stream then was extracted and analyzed via GC/FID (gas chromatography equipped with a flame ionization detector).

### 2.1. Influence of Tag Position of Enzyme for Reactor

**Figure 2:**
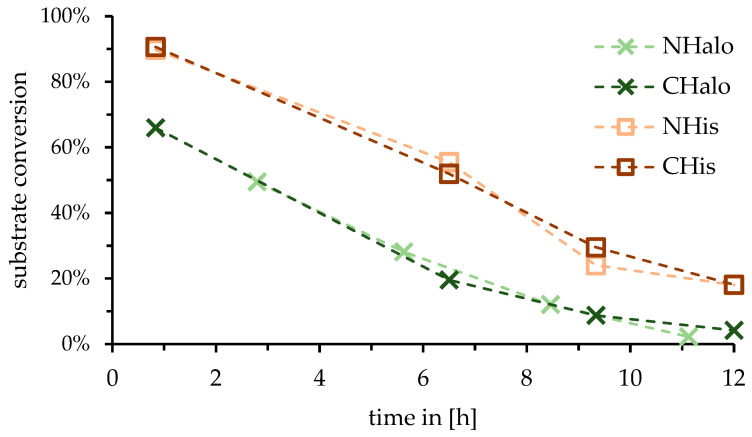
Change in substrate conversion over time by continuous usage for the reaction of hexanal (**1**) and acetaldehyde (**2**) towards 3-hydroxyoctanal (**3**) catalyzed via the DERA reactors. (Hexanal (**1**) as observed substrate.) Light-green cross: reactor with N-terminal HaloTag^®^-DERA (NHalo). Dark-green cross: reactor with C-terminal HaloTag^®^-DERA (CHalo). Light-brown square: reactor with N-terminal HisTag-DERA (NHis). Dark-brown square: reactor with C-terminal HisTag-DERA (CHis). Outliers are not shown (compare Supplementary Figure S3).

As most chosen immobilization methods rely on specific tags, the influence of the tag position on the assessed reaction was tested. For the N- and C-terminal HaloTag^®^ variant (NHalo, CHalo) and the N- and C-terminal HisTag variant of the DERA (NHis, CHis), no difference in performance could be observed (Figure 2). As C-terminal variants could be expressed slightly better (refer Supplementary Figures S1 and S2), further studies focused on these.

### 2.2. Initial Performance and Stability of Reactors

**Figure 3:**
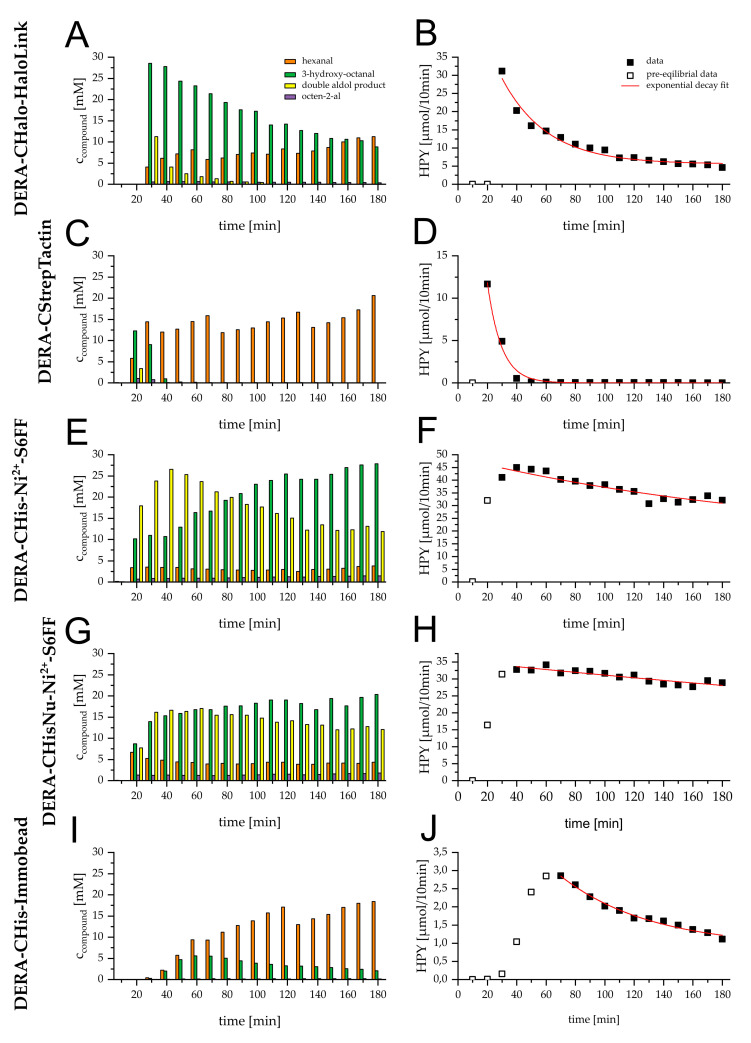
Left: Reactor (350 µL) performance based on different immobilizates for the reaction of 200 mm hexanal (**1**) with 600 mm acetaldehyde (**2**) in 100 mm triethanolamine (TEA) buffer pH 7 with 50 µL min^−1^. 10 min of product stream was extracted and analyzed via GC/FID. Content of samples is shown over time: orange; hexanal (**1**) green; (*R*)-3-hydroxy-octanal (**3**) purple; octen-2-al (**4**) yellow; hemicaetal **6**. Right: Filled squares show hypothetical product yield (HPY) for 10 min timeframe of 3-hydroxy-octanal (**3**) assuming all reactor activity was only used to conduct the first aldol reaction and no aldol condensation happened. Outlined squares: fractions of the reactor’s equilibration, excluded from fit. Red line shows exponential decay fit of HPY 10 min^−1^ after equilibration (for fit parameters, see Supplementary Table S1). (**A**,**B**): Product stream of DERA-CHalo-HaloLink^®^ reactor. (**C**,**D**): Product stream of DERA-CStrep-StrepTactin™ reactor. (**E**,**F**): Product stream of DERA-CHis-Ni^2+^-S6FF (IMAC Sepharose^®^ 6 FastFlow) reactor. (**G**,**H**): Product stream of DERA-CHisNu-Ni^2+^-S6FF reactor. (**I**,**J**): Product stream of DERA-CHis-Immobead reactor.

To compare the immobilization strategies, each type of reactor was observed for three hours with respect to product stream composition. The obtained profiles can be seen in Figure 3, left. All graphs show an initial equilibration phase. This phase was between 10 min and 30 min for all reactor types, except for the Immobead-based reactor. The theoretical dead volume of the system (590 µL) partially explained this equilibration phase. For the Immobead-based reactor, the time needed to reach the plateau was 50 to 60 min.

The reaction conditions were kept identical for a direct comparison between all reactors. However, DERA reactors tend to overshoot to a second aldol reaction, yielding the hemiacetal (**6**). As the reaction conditions were kept constant and the reaction was not optimized yet, this overshoot activity still needed to be accounted to a reactor’s activity. To use the side product formation in the evaluation, the side product amounts were weighted rationally and summed up to a hypothetical product yield (HPY) for the samples collected over 10 min. The trend of this yield resembled exponential decay, so a model was fitted to the data (see Figure 3, right). With those functions in hand, the half-life of the reactors could be determined. The determined parameters can be seen in Supplementary Table S1, and the calculated HPYs can be seen in Table 1.

The metal ion affinity (MIA)-based reactors started with the highest hypothetical yields: 4.5 µmol min^−1^ for the MIA immobilizate originating from the standard HisTag (DERA-CHis-Ni-S6FF) and 3.4 µmol min^−1^ for the variant with the modified HisTag (DERA-CHisNu-Ni-S6FF). The HaloTag^®^-based reactor showed a comparable hypothetical yield of 2.9 µmol min^−1^, whereas the Immobead-based reactor had the lowest hypothetical yield with 0.3 µmol min^−1^. The StrepTag-based reactor peaked at 1.2 µmol min^−1^.

In terms of the expected yield, the MIA-based reactors showed the highest hypothetical amount of product with 1050 µmol and 1300 µmol (DERA-CHis-Ni-S6FF and DERA-CHisNu-Ni-S6FF, respectively) upon reaching the half-life. The HaloTag^®^-based reactor was expected to produce ~60 µmol until its half-life. The Immobead-based reactor and the StrepTag based reactor should have yielded 16 µmol and 6 µmol. Normalized to the reactor half-life, the HaloTag^®^-based and the two MIA-based reactors performed comparably, with 2.1 µmol min^−1^ (DERA-Chalo-Link), 3.1 µmol min^−1^ (DERA-CHis-Ni-S6FF) and 2.4 µmol min^−1^ (DERA-CHisNu-Ni-S6FF) of hypothetical expected yield.

### 2.3. Time Demand from Harvested Culture to Ready Reactor

Due to the demanding nature of the conducted reaction, new reactors need to be prepared constantly to maintain productivity. As the tested immobilization methods differed, the time needed to prepare the immobilizates also varied.

In general, reactor preparation starts with the cultivation of recombinant expression strains, including the precultivation and harvesting of cells. These steps are identical for all investigated products. Therefore, the cell pellet production is not considered part of the reactor preparation. The actual preparation of a reactor involves three steps: cell lysis, target protein isolation, and immobilization. Cell lysis was conducted using 5 min to 15 min ultrasonication treatment of resuspended pellets, lysate separation from cell debris via centrifugation for 40 min and short filtering through a polyethersulfone membrane, corresponding to one hour in total. The target protein isolation happens inherently during immobilization for the chosen methods except in epoxy-based immobilization, where the immobilization is not guided by a specific affinity and would target all proteins in a solution. For this method, purification via immobilized metal ion affinity chromatography (IMAC) was conducted, adding two hours to this approach.

**Figure 4:**
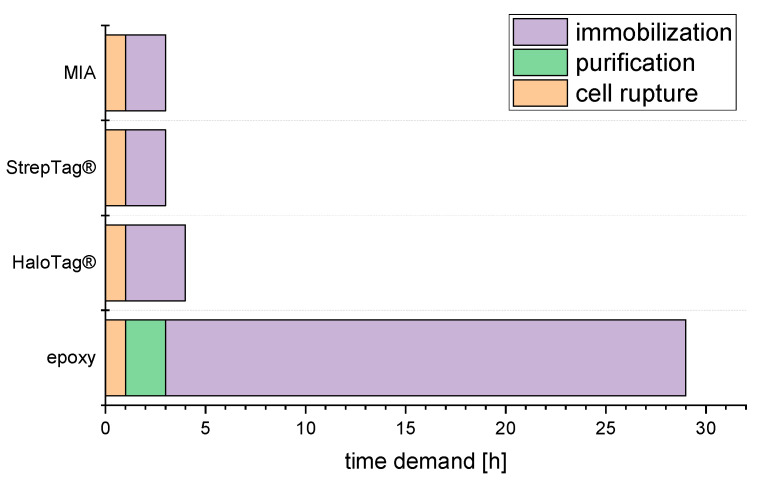
Time demand for reactor preparation of investigated immobilization approaches. MIA: metal ion affinity.

The immobilization step took different amounts of time, depending on the applied immobilization method. For the MIA-based and the StrepTag-based approach, 3.5 mL (~10 column volume (CV)) of filtered lysate was pumped through a packed column at 50 µL min^−1^ (roughly 0.15 CV min^−1^). For HaloTag^®^-based immobilization, 3.5 mL of filtered lysate was pumped through a packed column at 30 µL min^−1^ according to Döbber et al. [30]. The Immobead 150P epoxy functionalized resin needed to be incubated with a protein solution for 24 h under gentle agitation. Regardless of the immobilization method, gentle and thorough purging with buffer was carried out to wash out unbound and loosely bound protein. Therefore, the time demand for the MIA- and StrepTag-based approach was ~2 h; for HaloTag^®^-based, ~3 h; and for Immobead-based immobilization, ~26 h. The total time demand is visualized in Figure 4.

## 3. Discussion

### 3.1. Influence of Tag Position of Enzyme for Reactor

To investigate the influence of the tag position on the particular DERA reactor performance, both the Halo-Tag^®^ and the HisTag were fused to the DeoC*_EC_*-C47M-A95C N- and C-terminus, respectively. Reactors prepared with these variants showed no difference in conversion to the chosen model reaction (see Figure 2). This seemingly contradicts the work of Schulte et al. [31], who showed that the C-terminus of DERA significantly determines its activity. However, the used activity test utilizes the natural substrate in a retro-aldol reaction, while in the present investigation, an aldol reaction with unnatural substrate was observed.

In any case, C-terminally modified DERA variants were expressed slightly better than N-terminally modified variants. As no difference was observed and enough protein could be obtained, no further investigation was conducted.

### 3.2. Initial Performance and Stability of Reactors

In general, the activity of the reactors dropped very fast during the experiments. This can be attributed to the chosen substrate hexanal (**1**) having an amphiphilic character. Amphiphilic molecules may unfold proteins. The hydrophilic head may interact with a protein surface. The hydrophobic tail may interact with a protein core. This eases the unfolding of protein [32,33].

The initial equilibration phase of the reactor systems was not uniform for all reactors, although the dead volume (~590 µL) was the same. At a constant flow rate of 50 µL min^−1^, the theoretical equilibration time was roughly 12 min. The equilibration phase here was defined as the timeframe prior to the highest activity time point for a reactor. The equilibration phase of the DERA-CStrep-StrepTactin™ reactor was within this range. The MIA-based reactor system’s and the DERA-CHalo-HaloLink^®^ reactor’s equilibration phases were more in the range of 20 min to 30 min (see Figure 3). This was topped by the Immobead-based reactor system, with an equilibration phase of 50 min to 60 min. These differences can be explained by the chromatographic interaction of the observed compounds with the respective protein carrier materials. The HaloLink^®^ resin, the StrepTactin™ Sepharose™ and the IMAC Sepharose™ 6 FastFlow materials are based on cross-linked agarose. The hydrophilic agarose matrix in combination with aqueous buffer may lead to a small chromatographic effect. Immobeads, on the other hand, are based on methacrylamide, which is more hydrophobic. Therefore, the matrix interacts more strongly with the components than with the buffer, leading to retention. The DERA-CStrep-StrepTactin™ reactor therefore presumably shows an equilibrium phase that is close to that of a reactor without matrix effects, as this reactor type is particularly affected by rapid deactivation. The measurement here was carried out via the products formed, so that with a high deactivation rate, the early measurement points appeared apparently higher, and chromatographic effects could thus hardly be detected.

In Figure 3, graph C and I show the waviness of the hexanal (**1**) concentration in a 60 min period. This is an artifact due to sample handling. The sample preparation for measurements was conducted in batches of six. Therefore, the oldest sample in such a batch had already stood for an hour in the autosampler rack. Hexanal is somewhat volatile (1.5 kPa (25 °C) vapor pressure) [34], leading to material evaporation. To a smaller extent, this is true for the desired product (*R*)-3-hydroxy-octanal (**3**), also visible in Figure 3, graph E. The vapor pressure for (*R*)-3-hydroxy-octanal (**3**) is assumed to be smaller than that from hexanal (**1**) due to the additional hydroxy functionality; hence, this batch effect is not that prominent for this compound.

As the MIA-based reactors worked relatively stable within the observed period, the loss of activity showed rather linear behavior. To keep the comparability, exponential decay was fitted, regardless. However, as the data did not resemble exponential decay, the fit parameters inherited a high error (see Supplementary Table S1). Some models did not converge to zero activity (see Supplementary Table S1). This could be a measuring artifact, or the protein was stabilized on the matrix.

From the tested immobilization approaches, the MIA-based methods worked best for the DERA. In general, if an immobilized catalyst is demanded, the MIA-based immobilization is a good first choice. Not only did the MIA-based reactors outperform the others in this direct comparison with respect to activity, the HisTag is already commonly fused to investigated proteins, also. In a case in which the protein–matrix conjugate is not as stable as observed here (leaching problem), HaloTag^®^-based immobilization is a good second choice.

### 3.3. Time demand from Harvested Culture to Ready Reactor

Among the investigated immobilization methods, the epoxy-based immobilization reactor preparation takes by far the most time, taking roughly 29 h (see Figure 4). Additionally, prolonged incubation for the actual epoxide opening reaction may be fatal for less stable proteins. For purified DERA, just a minor loss in activity was observed. However, protection measures may need to be applied for other proteins. There was no big difference between the other methods, which required three to four hours. Therefore, quick preparation for a replacement reactor can easily be scheduled. In terms of reactor preparation time demand, we conclude that epoxy-based immobilization methods should not be used, although they form a covalently bound catalyst.

## 4. Materials and Methods

### 4.1. Chemicals

The chemicals used in this work were commercially purchased from *Merck* (*Merck KGaA*, Darmstadt, Germany) and *TCI* (*TCI Deutschland GmbH*, Eschborn, Germany).

### 4.2. Software

OriginPro 2019 (64-bit) v 9.6.0.172 (*OriginLab Corporation*, Northampton, MA, USA).

### 4.3. Methods

#### 4.3.1. Cloning of DERA Mutants

The 2-deoxy-d-ribose-5-phosphate aldolase (DERA) mutants were created over several steps by the combination of restriction–ligation cloning, *Gibson* assembly, overlap extension assembly and round-the-horn mutagenesis [35,36]. Oligonucleotides were synthesized by *Merck KGaA*. Sequence identity was checked via *GATC SupremeRun* sequencing (*Eurofins Genomics Germany GmbH*, Ebersberg, Germany).

#### 4.3.2. Protein Production

Chemically competent *E. coli* BL21(DE3) were transformed with the respective plasmid. A colony of a transformed clone was used to inoculate a 25 mL LB_Amp_ (lysogeny broth) [37] medium overnight culture (100 µg mL^−1^ ampicillin; in 100 mL *Erlenmeyer* flask at 37 °C with 120 rpm agitation). The entire culture was used to inoculate 1 L TB_Amp_ medium (100 µg mL^−1^ Ampicillin) [38] in a 3 L *Fernbach* flask. The culture was incubated at 37 °C with 120 rpm until an optical density (OD_600 nm_) of 0.3 was reached. Then, 100 µm of isopropyl-β*-*d-thiogalactopyranosid (IPTG) was added for the induction of expression. After a 30 min cold shock on ice, the culture was incubated at 25 °C with 120 rpm for 24 h. The cells were then harvested by centrifugation at 4 °C with 8200 rcf for 30 min. The DERA-containing cell pellet was stored at −20 °C until use.

#### 4.3.3. Immobilization of DERA Variants

About 1 g of DERA-containing cell pellet was resuspended with 4 mL g^−1^ in 100 mm triethanolamine (TEA) buffer with a pH of 7. The resuspension was then ultrasonicated (*BANDELIN SONOPLUS* with a *SONOPLUS* KE76 cone tip [*BANDELIN electronic GmbH & Co. KG,* Berlin, Germany], twotimes to fourtimes 5 min, 50% cycle, 35% amplitude in 50 mL reaction vessel) while chilled on ice. The lysis efficiency was determined by measuring OD_600 nm_ prior to and after sonication and was calculated this way:

(1) $$lysis\;efficiency=1-\frac{OD_{post}}{OD_{pre}}$$

A lysis efficiency below 75% was avoided. The crude cell extract was then centrifuged (4 °C, 7000 rcf, 40 min) and filtered with a 0.2 µm polyethersulfone membrane.

For the HaloTag^®^-based reactor, an *Omnifit*^®^ (*Diba industries Inc.*, Cambridge, United Kingdom) column (3 mm Ø by 50 mm) was tightly packed with HaloLink^®^-Resin (*Promega GmbH*, Walldorf, Germany) to the total volume of ~350 µL. The resin then was washed with 10 mL of deionized water and pumped with a syringe pump *kdS Legato 100* (*kdScientific Inc.,* Holliston, Massachusetts, *USA*) at a flow rate of 1 mL min^−1^. While water was pumped through, a turned-on vortex was placed close to the laboratory stand to rattle out air bubbles from the column. Then, 5 mL of 100 mm TEA buffer with a pH of 7 was flown through at 350 µL min^−1^ to equilibrate the matrix material. Next, 3.5 mL of the filtered, DERA-containing lysate was pumped though the column at 30 µL min^−1^. This was followed by 1 mL of buffer at the same flow rate. Together with the flown-through 3.5 mL of lysate, this was considered as the flow-through sample. Finally, 5 mL of buffer was applied to wash the reactor at 200 µL min^−1^.

The protocol for metal-ion-affinity-based (MIA) reactors and StrepTag™-based reactors is the same as for HaloTag^®^-based reactor, except the flowrate for lysate loading, where 50 µL min^−1^ was used. For MIA reactors, IMAC Sepharose^®^ 6 FastFlow (*cytiva*, Marlborough, Massachusetts, USA) was tightly packed. After being washed with deionized water, 1 mL of 100 mm NiCl_2_ was pumped at 200 µL min^−1^ to charge the matrix with Ni^2+^ ions. Then, 5 mL of deionized water at 200 µL min^−1^ was used prior to the equilibration step. For StrepTag™-based reactors, the columns were packed with StrepTactin™ sepharose^®^ (*IBA Lifesciences GmbH*, Göttingen, Germany).

For immobilization on the epoxy group carrying Immobead 150P (*ChiralVision B.V.*, Den Hoorn, Netherlands), purified protein was needed. Therefore, 4 g of cell pellet containing DERA*_EC_* DM CHis was resuspended in 16 mL of 50 mm potassium phosphate (KP_i_) buffer with a pH of 7 and lysed as described above. A 5 mL HiTrap™ IMAC FF (*Cytiva*) was cyclically charged for 30 min at roughly 2 mL min^−1^ with a peristaltic P-1 pump (*Cytiva*). Then, 10 mL of KP_i_ buffer was applied to wash out unbound protein. Loosely bound protein was eluted by collecting ten fractions of 5 mL with KP_i_ buffer containing 20 mm imidazole. The protein of interest was eluted by raising the imidazole concentration to 250 mm (also collecting ten fractions of 5 mL, each). Next, 20 mL of 1 m imidazole-containing buffer was applied to elute all remaining protein. The fractions with highest specific activity were unified and concentrated two times for 5 min at 5440 rcf, 4 °C with *Vivaspin*^®^ concentrator (*Sartorius AG*, Göttingen, Germany) and desalted with PD-10 columns (*Cytiva*). Then, 550 mg of Immobead 150P was incubated with 4.5 mL of protein preparation for 24 h under gentle agitation. Then, the Immobeads were washed three times with 10 mL of buffer (100 mm TEA pH 7) and filled in the *Omnifit*^®^ column.

#### 4.3.4. DERA Activity Assay

The retro aldol activity with respect to the 2-deoxy-d-ribose-5-phospahte (DRP) cleavage of protein samples was measured for 25 min at 25 °C with the auxiliary proteins glycerol-3-phosphate dehydrogenase (GDH) and triosephosphate isomerase (TPI) by following the depletion of reduced nicotinamide-adenine-dinucleotide (NADH) at 340 nm with an *Infinite M1000pro* (*Tecan Group AG*, Männedorf, Switzerland) microtiter plate reader [6,39]. Per well, 5 µL of sample was placed in. The assay was started by adding 195 µL of assay mix containing 188,8 µL of 100 mm TEA buffer with a pH of 7, 2 µL of 40 mm DRP, 4 µL of 10 mm NADH and 0.2 µL of GDH/TPI-Mix (155 U mg^−1^ GDH, 1630 U mg^−1^ TPI). The protein concentration was determined by the *Bradford* assay [40].

#### 4.3.5. Initial performance Flow Experiment Setup

**Figure 5:**
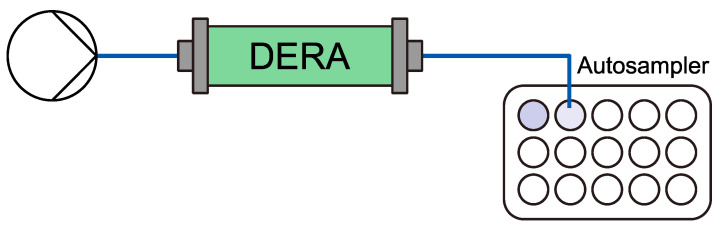
General flow setup. The reaction mixture containing hexanal (**1**) and acetaldehyde (**2**) in TEA puffer were pumped through a DERA-containing packed bed reactor. The product stream was collected afterwards by an autosampler.

200 mm hexanal (**1**) (above solubility, emulsion formed) and 600 mm acetaldehyde (**2**) were suspended in 100 mm TEA buffer with a pH of 7 and pumped with a *kdS Legato 100* (*KD Scientific Inc.*) syringe pump at 50 µL min^−1^ through a freshly prepared 350 µL reactor. After the reactor, a *FC 203B* fraction collector (*Gilson Inc*., Middleton, Wisconsin, USA) fractionated the stream in 500 µL (10 min) samples for 3 h. The setup scheme is depicted in Figure 5.

Then, 400 µL per fraction was extracted with two times 200 µL ethyl acetate with 2-phenylethanol of the defined concentrations. The organic phase was collected and dried with MgSO_4_•H_2_O. The sample was then analyzed by GC/FID (gas chromatography with a flame ionization detector) (*ThermoQuest TRACE-GC* [*Thermo Fisher Scientific*, Waltham, Massachusetts, USA]), FS-Hydrodex β-3P-capillary column (25 m 0.4 mm outer diameter, 0.25 mm inner diameter) (*Macherey-Nagel*, Düren, Germany), 1 µL sample injection with MTBE (methyl-*tert*-butylether). Temperature profile: 60 °C for 5 min; 5 °C min^−1^ until 200 °C; 200 °C for 5 min. The retention times were taken from *Bisterfeld* [41]. The compound concentrations were determined as described by *de Saint Laumer* et al. [42].

#### 4.3.6. Tag Position Dependent Performance

Reactor columns were treated with reaction mixture (200 mm hexanal (**1**) and 600 mm acetaldehyde (**2**) were suspended in 100 mm TEA buffer with a pH of 7) for 12 h at 50 µL min^−1^. A sample was collected for 40 min (2 mL). Then, 1 mL was extracted with two times 500 µL of ethyl acetate containing a defined concentration of 2-phenylethanol. The extract was dried with MgSO_4_•H_2_O and analyzed by GC/FID (*ThermoQuest TRACE-GC* [*Thermo Fisher Scientific]*), FS-Hydrodex β-3P-capillary column (25 m 0.4 mm outer diameter, 0 25 mm inner diameter) [*Macherey-Nagel*]) 1 µL sample injection with MTBE. Temperature profile: 60 °C for 5 min; 5 °C min^−1^ until 200 °C; 200 °C for 5 min.

#### 4.3.7. Data Evaluation

To calculate a hypothetical product yield, the products originating from productive conversions performed by the reactor were considered. These were the first aldol addition products—the desired product (**3**), the aldol condensate (**4**), a side product formed by the condensation of the desired product and the cyclized double aldol addition product (**6**), formed from a subsequent aldol addition from the desired product, also catalyzed by the reactor (refer to Figure 1). As kinetic investigations of the actual catalyst—the DERA—generally show a ratio of catalytic efficiency of the first aldol reaction and the second aldol reaction of ~1.95, the second reaction performed roughly half as well as the first reaction [43]. Therefore, the amount of cyclized double aldol addition product was weighted by 2.95. The exponential decay function

(2) $$y=y_{0}+A_{1}e^{-\frac{x}{t_{1}}}$$

was fitted with OriginPro (*OriginLab Corporation*) to the summed-up values per time batch. For the DERA-CHisNu-Ni^2+^-S6FF data, the y_0_ was set to zero to reach convergence. The half-life of the reactor and the expected total yield per reactor were then calculated based on the model.

## Figures and Tables

**Table 1:** Derived performance indicators for DERA reactors. DERA-CHalo-Link: DeoC*_EC_*-C47M-A95C with C-terminal HaloTag^®^ immobilized on HaloLink^®^-resin. CStrep-Tactin: DeoC*_EC_*-C47M-A95C with C-terminal StrepTag immobilized on StrepTactin™-Sepharose^®^. CHis-Ni-S6FF: DeoC*_EC_*-C47M-A95C with C-terminal HisTag immobilized on IMAC Sepharose^®^ 6 FastFlow tethered by Ni^2+^ ions. CHisNu-Ni-S6FF: DeoC*_EC_*-C47M-A95C with C-terminal HisTag with additional Cysteins immobilized on IMAC Sepharose^®^ 6 FastFlow tethered by Ni^2+^ ions. CHis-Immobead: DeoC*_EC_*-C47M-A95C with C-terminal HisTag immobilized on Immobead150P. Reactor size: ~350 µL. Peak yield observed directly after equilibration phase. Half-life: time until peak yield is halved, starting from peak yield time point. Exp. yield: hypothetical amount of substance produced upon reaching reactor’s half-life starting from peak yield time-point. Av. yield: expected hypothetical yield averaged over half-life.

	Peak Yield	Half-Life	Exp. Yield	Av. Yield
	HPY/t	τ_1/2_	HPY	HPY/t
	µmol/min	min	µmol	µmol/min
DERA-CHalo-Link	2.92	30	61.5	2.07
DERA-CStrep-Tactin	1.18	7	5.7	0.85
DERA-CHis-Ni-S6FF	4.48	339	1050	3.09
DERA-CHisNu-Ni-S6FF	3.36	538	1300	2.43
DERA-CHis-Immobead	0.28	80	15.9	0.20

## Data Availability

The raw data for the presented results are available upon request from the authors.

## Supplementary Materials

The following supporting information can be downloaded at: https://www.mdpi.com/article/10.3390/molecules27196483/s1: Procedure for SDS-PAGE according to *Schägger* et al. and *Kang* et al. [44,45], Supplementary Figure S1 and Supplementary Figure S2: SDS-PAGEs for protein expression control, Supplementary Figure S3: Full data for tag-position dependent reactor activity, Supplementary Table S1: Fitting parameters for exponential decay fit and quality of fit, including corrected R^2^ [46], Supplementary Figure S4: Plasmid map of used vector plasmid, DNA sequences of used mutants and aminoacid sequences of the respective variants of *E. coli* DERA DeoC [47].

## Sample Availability

Plasmids harboring the genes for the used enzymes in this study are available upon request.
