# Supplementary material for: Simple Enzyme Immobilization for Flow Chemistry? An Assessment of Available Strategies for an Acetaldehyde-Dependent Aldolase
Source: Molecules. 2022 Oct 1;27(19):6483. doi: 10.3390/molecules27196483 (PMC9570893; doi:10.3390/molecules27196483)
Supplement: Supplementary file 1 [file molecules-27-06483-s001.zip › molecules-1911392-supplementary.pdf]

molecules

Supporting Information for

Simple Enzyme Immobilization for Flow Chemistry? An Assessment of Available Strategies for an Acetaldehyde-Dependent Aldolase

Martin Wäscher [1], Thomas Classen [2] and Jörg Pietruszka [1,2,*]

[1] Institute for Bioorganic Chemistry; Heinrich Heine University Düsseldorf, 40225 Düsseldorf, Germany
[2] Institute for Bio- and Geosciences 1: Bioorganic Chemistry, Forschungszentrum Jülich, 52425 Jülich, Germany
* Correspondence: j.pietruszka@fz-juelich.de; Tel.: +49-(0)2461-61-4158

SDS-PAGE

SDS-PAGE samples have been prepared by diluting *ad* 60 μL deionized water according to empirical equation

$$V_{sample} = \frac{375\ \mu L}{OD_{600}} \quad (1)$$

for cell containing samples, or equation

$$V_{sample} = \frac{72\ mg\ mL^{-1}}{protein\ amount\ in\ [mg\ mL^{-1}]} \quad (2)$$

for protein solutions. 15 μL 5x SDS-PAGE sample buffer (10 % w/v SDS, 40 % v/v glycerol, 0.5 % w/v bromophenol blue, 500 mM dithiothreitol in 500 mM Tris/HCl buffer, pH 6.5) were added and the mixture was vortexed. Before application on gel, the samples were heated for 10 min at 95 °C.

0.75 mm gels were prepared with 4 % collection phase and 10 % separation phase. Electrophoresis was carried out at 190 V, 3 Amax for 40 min driven by a *PowerPac HC* (*BioRad*) according to *Schägger et al.* [1]. Staining was conducted as described by *Kang et al* [2]. The result was documented with an *EOS 1000D* (*Canon*).

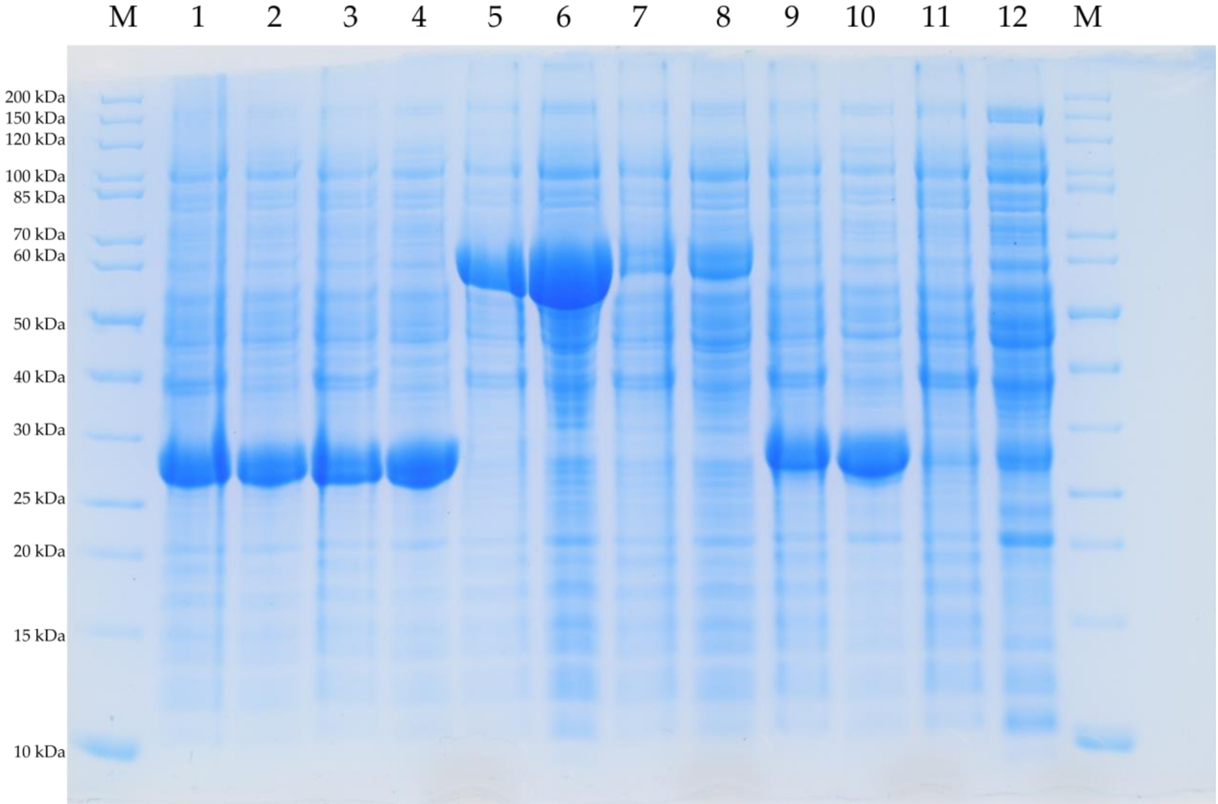

Supplementary Figure S1. SDS-PAGE for protein expression control. **M:** 5 µL Protein Marker: PageRuler™ unstained protein ladder (ThermoScientific) contains Proteins of size: 200 kDa, 150 kDa, 120 kDa, 100 kDa, 85 kDa, 70 kDa; 60 kDa, 50 kDa, 40 kDa, 30 kDa, 25 kDa, 20 kDa, 15 kDa, 10 kDa. **1:** 15 µL sample from deoC-EC-DM-CHis expression (pellet resuspension), calculated molecular weight (MW_c): 28.9 kDa. **2:** 15 µL sample from DeoC-EC-DM-CHis preparation (clear cell-free extract), MW_c: 28.9 kDa. **3:** 15 µL sample from deoC-EC-DM-NHis expression (pellet resuspension), MW_c: 28.9 kDa. **4:** 15 µL sample from DeoC-EC-DM-NHis preparation (clear cell-free extract), MW_c: 28.9 kDa. **5:** 15 µL sample from deoC-EC-DM-CHalo expression (pellet resuspension), MW_c: 63.6 kDa **6:** 15 µL sample from DeoC-EC-DM-CHalo preparation (clear cell-free extract), MW_c: 63.6 kDa. **7:** 15 µL sample from deoC-EC-DM-NHalo expression (pellet resuspension), MW_c: 63.8 kDa. **8:** 15 µL sample from DeoC-EC-DM-NHalo preparation (clear cell-free extract), MW_c: 63.8 kDa. **9:** 15 µL sample from deoC-EC-DM-CHisNu expression (pellet resuspension), MW_c: 29.2 kDa. **10:** 15 µL sample from DeoC-EC-DM-NNuHis preparation (clear cell-free extract), MW_c: 29.2 kDa. **11:** 15 µL sample from deoC-EC-DM-NNuHis expression (pellet resuspension), MW_c: 29.2 kDa. **12:** 15 µL sample from DeoC-EC-DM-NNuHis preparation (clear cell-free extract), MW_c: 29.2 kDa.

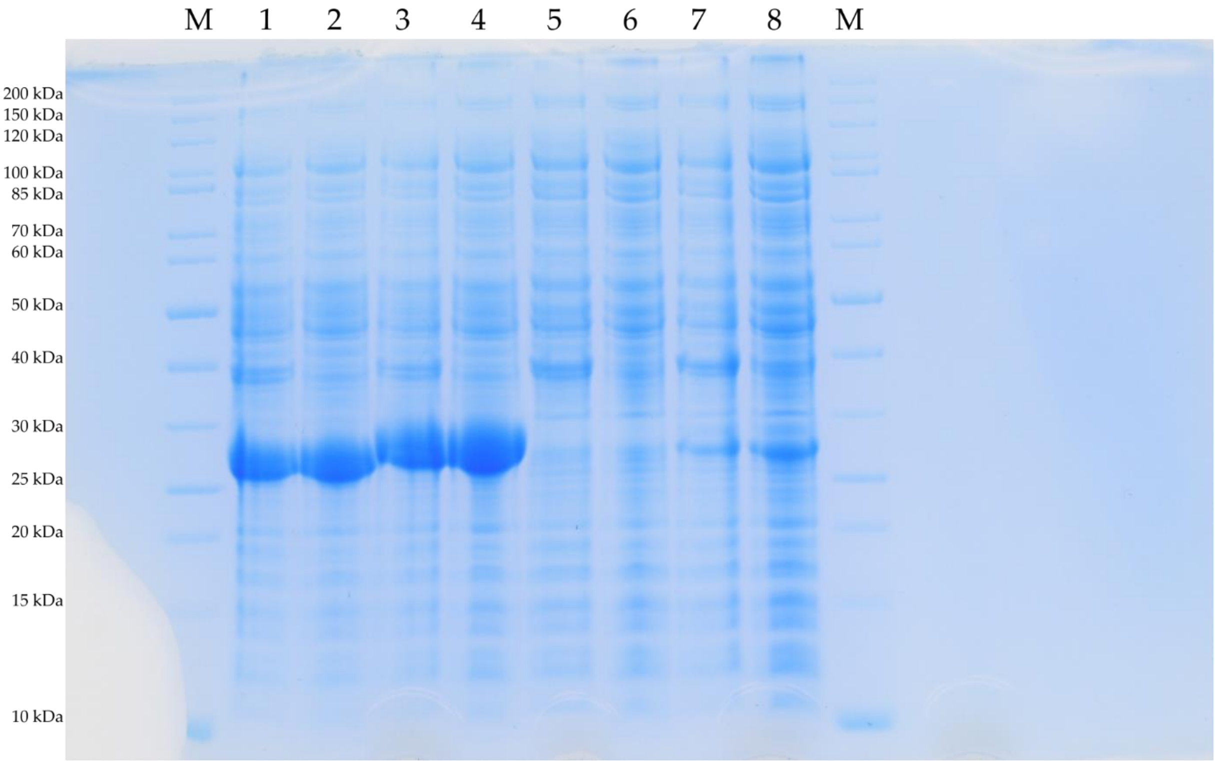

Supplementary Figure S2. SDS-PAGE for protein expression control. **M:** 5 µL Protein Marker: PageRuler™ unstained protein ladder (ThermoScientific) contains Proteins of size: 200 kDa, 150 kDa, 120 kDa, 100 kDa, 85 kDa, 70 kDa; 60 kDa, 50 kDa, 40 kDa, 30 kDa, 25 kDa, 20 kDa, 15 kDa, 10 kDa. **1:** 15 µL sample from deoC-EC-DM-CNu expression (pellet resuspension), calculated molecular weight (MW_c): 28.4 kDa. **2:** 15 µL sample from DeoC-EC-DM-CNu preparation (clear cell-free extract), MW_c: 28.4 kDa. **3:** 15 µL sample from deoC-EC-DM-CStrep expression (pellet resuspension), MW_c: 29.3 kDa. **4:** 15 µL sample from DeoC-EC-DM-CStrep preparation (clear cell-free extract), MW_c: 29.3 kDa. **5:** 15 µL sample from deoC-EC-DM-NNu expression (pellet resuspension), MW_c: 28.4 kDa **6:** 15 µL sample from DeoC-EC-DM-NNu preparation (clear cell-free extract), MW_c: 28.4 kDa. **7:** 15 µL sample from deoC-EC-DM-NStrep expression (pellet resuspension), MW_c: 29.3 kDa. **8:** 15 µL sample from DeoC-EC-DM-NStrep preparation (clear cell-free extract), MW_c: 29.3 kDa.

Reactor stability - full data

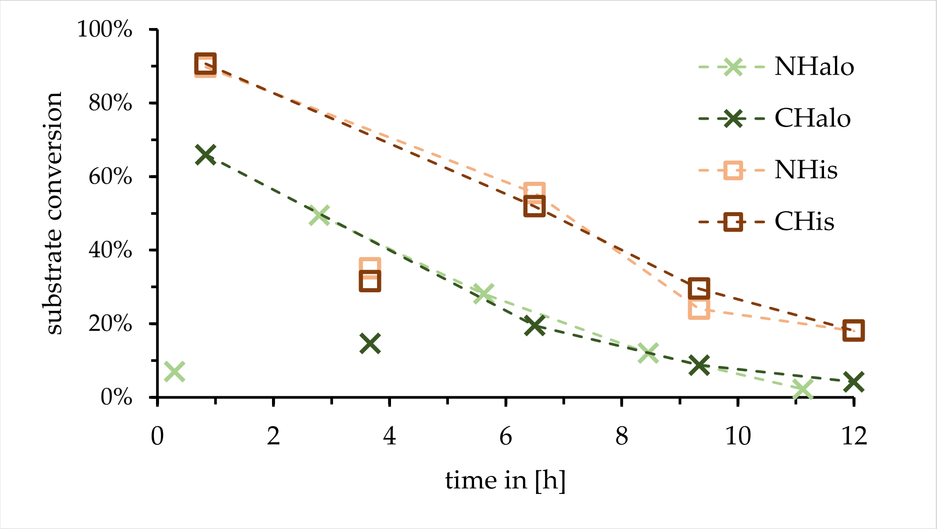

Supplementary Figure S3. Change in substrate conversion over time by continuous usage for the reaction of hexanal (**1**) and acetaldehyde (**2**) towards 3-hydroxyoctanal (3) catalyzed by the DERA reactors. (hexanal (1) as observed substrate). Light-green cross: reactor with N-terminal HaloTag®-DERA (NHalo). Dark-green cross: reactor with C-terminal HaloTag®-DERA (CHalo). Light brown square: reactor with N-terminal HisTag-DERA (NHis). Dark-brown square: reactor with C-terminal HisTag-DERA (CHis).

Fit parameters

Supplementary Table S1. DERA-CHalo-Link: DeoC$_{EC}$-C47M-A95C with C-terminal HaloTag® immobilized on HaloLink®-resin. CStrep-Tactin: DeoC$_{EC}$-C47M-A95C with C-terminal StrepTag immobilized on StrepTactin™-Sepharose®. CHis-Ni-S6FF: DeoC$_{EC}$-C47M-A95C with C-terminal HisTag immobilized on IMAC Sepharose® 6 FastFlow tethered by Nickel ions. CHisNu-Ni-S6FF: DeoC$_{EC}$-C47M-A95C with C-terminal HisTag with additional cysteins immobilized on IMAC Sepharose® 6 FastFlow tethered by nickel ions. CHis-Immobead: DeoC$_{EC}$-C47M-A95C with C-terminal HisTag immobilized on Immobead150P. Reactor size: ~350 µL.

	Fit-parameters			Fit-Quality	
	limit t→∞ y0 [µmol/10 min]	initial t=0 A_1 [µmol/10 min]	decay rate factor t_1 [x10 min]	R^2	corr. R^2 ‡
DERA-CHalo-Link	5.6 ± 0.6	62.4 ± 7.6	30.9 ± 3.4	0.97	0.97
DERA-CStrep-Tactin	0.0 ± 0.1	96.1 ± 14.9	9.6 ± 0.7	0.99	0.99
DERA-CHis-Ni^{2+}-S6FF	15.7 ± 28.5	33.1 ± 25.8	230.9 ± 308.3	0.85	0.83
DERA-CHisNu-Ni^{2+}-S6FF	0 ± 174	35.4 ± 172.5	776.9 ± 4392	0.81	0.78
DERA-CHis-Immobead	0.9 ± 0.2	6.1 ± 0.8	61.6 ± 10.4	0.99	0.98

‡: corrected R^2 considers degrees of freedom of fit. Please refer to *OriginLab*-manual for detailed calculation [3].

Sequences

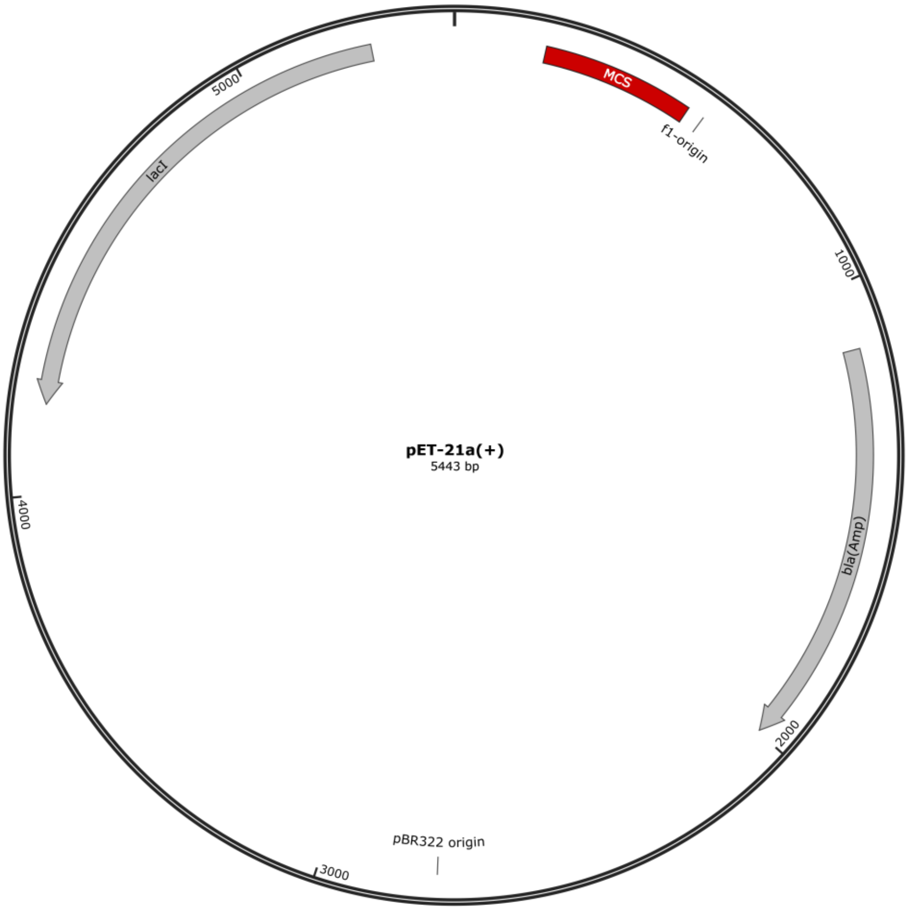

Supplementary Figure S4. Plasmid map of pET-21a(+)

pET21a(+)-MCS

```
GATC         Plasmid features

   1 AGATCTCGAT CCCGCGAAAT TAATACGACT CACTATAGGG GAATTGTGAG CGGATAACAA TTCCCCTCTA GAAATAATTT
                         T7-Promoter         lac-Operator
  81 TGTTTAACTT TAAGAAGGAG ATATACATAT GGCTAGCATG ACTGGTGGAC AGCAAATGGG TCGCGGATCC GAATTCGAGC
                                 T7-Tag
 161 TCCGTCGACA AGCTTGCGGC CGCACTCGAG CACCACCACC ACCACCACTG AGATCCGGCT GCTAACAAAG CCCGAAAGGA
                                    His-Tag
 241 AGCTGAGTTG GCTGCTGCCA CCGCTGAGCA ATAACTAGCA TAACCCCTTG GGGCCTCTAA ACGGGTCTTG AGGGGTTTTT
                               T7-terminator
 321 TG
```

DeoC reference sequence

UniProt accession number: P0A6L0 [4]

pET21a-deoC-EC-DM-NHis_MCS-frame

```
GATC         Plasmid features
GATC         deoC-gene
GATC         gene modifications
GATC         HisTag

   1 AGATCTCGAT CCCGCGAAAT TAATACGACT CACTATAGGG GAATTGTGAG CGGATAACAA TTCCCCTCTA GAAATAATTT
                         T7-Promoter          lac-Operator
  81 TGTTTAACTT TAAGAAGGAG ATATACATAT GCATCACCAT CACCACCATG CTAGCACTGA TCTGAAAGCA AGCAGCCTGC
                                      HisTag              N-flank
 161 GTGCACTGAA ATTGATGGAC CTGACCACCC TGAATGACGA CGACACCGAC GAGAAAGTGA TCGCCCTGTG TCATCAGGCC
 241 AAAACTCCGG TCGGCAATAC CGCCGCTATC ATGATCTATC CTCGCTTTAT CCCGATTGCT CGCAAAACTC TGAAAGAGCA
                                  C47M
 321 GGGCACCCCG GAAATCCGTA TCGCTACGGT AACCAACTTC CCACACGGTA ACGACGACAT CGACATCGCG CTGGCAGAAA
 401 CCCGTGCGGC AATCTGCTAC GGTGCTGATG AAGTTGACGT TGTGTTCCCG TACCGCGCGC TGATGGCGGG TAACGAGCAG
                 A95C
 481 GTTGGTTTTG ACCTGGTGAA AGCCTGTAAA GAGGCTTGCG CGGCAGCGAA TGTACTGCTG AAAGTGATCA TCGAAACCGG
 561 CGAACTGAAA GACGAAGCGC TGATCCGTAA AGCGTCTGAA ATCTCCATCA AAGCGGGTGC GGACTTCATC AAAACCTCTA
 641 CCGGTAAAGT GGCTGTGAAC GCGACGCCGG AAAGCGCGCG CATCATGATG GAAGTGATCC GTGATATGGG CGTAGAAAAA
 721 ACCGTTGGTT TCAAACCGGC GGGCGGCGTG CGTACTGCGG AAGATGCGCA GAAATATCTC GCCATTGCAG ATGAACTGTT
 801 CGGTGCTGAC TGGGCAGATG CGCGTCACTA CCGCTTTGGC GCTTCCAGCC TGCTGGCAAG CCTGCTGAAA GCGCTGGGTC
 881 ACGGCGACGG TAAGAGCGCC AGCAGCTACG GATCCTGACT CGAGCACCAC CACCACCACC ACTGAGATCC GGCTGCTAAC
                               C-flank         His-Tag
 961 AAAGCCCGAA AGGAAGCTGA GTTGGCTGCT GCCACCGCTG AGCAATAACT AGCATAACCC CTTGGGGCCT CTAAACGGGT
                                             T7-Terminator
1041 CTTGAGGGGT TTTTTG
```

DeoC-EC-DM-NHis AA-sequence *“NHis”*

```
   1 MHHHHHHAST DLKASSLRAL KLMDLTTLND DDTDEKVIAL CHQAKTPVGN TAAIMIYPRF IPIARKTLKE QGTPEIRIAT
  81 VTNFPHGNDD IDIALAETRA AICYGADEVD VVFPYRALMA GNEQVGFDLV KACKEACAAA NVLLKVIIET GELKDEALIR
 161 KASEISIKAG ADFIKTSTGK VAVNATPESA RIMMEVIRDM GVEKTVGFKP AGGVRTAEDA QKYLAIADEL FGADWADARH
 241 YRFGASSLLA SLLKALGHGD GKSASSYGS
```

pET21a-deoC-EC-DM-NHalo_MCS-frame

```
GATC        Plasmid features
GATC        deoC-gene
GATC        gene modifications
GATC        linker-sequence
GATC        HaloTag

   1 AGATCTCGAT CCCGCGAAAT TAATACGACT CACTATAGGG GAATTGTGAG CGGATAACAA TTCCCCTCTA GAAATAATTT
                          T7-Promoter           lac-Operator
  81 TGTTTAACTT TAAGAAGGAG ATATACATAT GGCAGAAATT GGTACGGGAT TTCCGTTTGA CCCGCATTAT GTGGAGGTTC
 161 TGGGTGAACG CATGCACTAC GTGGATGTTG GTCCGCGCGA TGGCACACCG GTGCTGTTTC TGCATGGTAA TCCGACCTCC
 241 AGCTATGTTT GGCGCAACAT TATTCCGCAT GTCGCCCCAA CGCATCGCTG TATTGCCCCA GATCTCATTG GCATGGGCAA
 321 AAGCGACAAA CCGGATTTGG GCTACTTCTT CGACGATCAC GTACGGTTTA TGGACGCCTT TATCGAGGCT CTGGGACTGG
                                                                           C-->G (silent)
 401 AGGAAGTAGT GCTGGTTATT CATGACTGGG GCTCTGCATT AGGCTTTCAC TGGGCTAAAC GGAACCCAGA ACGCGTCAAG
 481 GGGATTGCCT TCATGGAGTT CATCCGTCCG ATTCCGACCT GGGATGAATG GCCCGAATTT GCCCGTGAAA CCTTTCAGGC
 561 GTTTCGTACC ACGGATGTTG GCCGTAAGCT CATCATCGAC CAAAACGTGT TCATTGAGGG CACTCTTCCC ATGGGAGTAG
 641 TGCGTCCTTT AACCGAAGTC GAGATGGACC ACTATCGCGA ACCCTTCCTG AATCCGGTTG ATCGCGAACC GCTGTGGCGC
 721 TTCCCGAATG AGCTGCCTAT TGCTGGTGAA CCGGCGAATA TCGTGGCACT TGTGGAAGAA TACATGGATT GGCTGCATCA
 801 GAGTCCAGTC CCTAAGCTGT TGTTTTGGGG TACACCTGGC GTGTTGATTC CGCCTGCAGA AGCTGCTCGC TTAGCGAAAA
 881 GCTTGCCCAA CTGCAAAGCG GTCGATATTG GGCCAGGTCT GAACCTGTTA CAGGAGGATA ACCCGGATCT GATCGGGAGT
 961 GAAATCGCGC GTTGGCTGTC AACTCTGGAA ATCTCGGGTC TTGCAGAAGC AGCGGCCAAA GAAGCTGCGG CCAAAGAGGC
                                                 4xEAAAK-linker
1041 AGCCGCGAAA GAAGCAGCGG CGAAAATGGC TAGCACTGAT CTGAAAGCAA GCAGCCTGCG TGCACTGAAA TTGATGGACC
                                N-flank
1121 TGACCACCCT GAATGACGAC GACACCGACG AGAAAGTGAT CGCCCTGTGT CATCAGGCCA AAACTCCGGT CGGCAATACC
1201 GCCGCTATCA TGATCTATCC TCGCTTTATC CCGATTGCTC GCAAAACTCT GAAAGAGCAG GGCACCCCGG AAATCCGTAT
             C47M
1281 CGCTACGGTA ACCAACTTCC CACACGGTAA CGACGACATC GACATCGCGC TGGCAGAAAC CCGTGCGGCA ATCTGCTACG
                                                                                 A95C
1361 GTGCTGATGA AGTTGACGTT GTGTTCCCGT ACCGCGCGCT GATGGCGGGT AACGAGCAGG TTGGTTTTGA CCTGGTGAAA
1441 GCCTGTAAAG AGGCTTGCGC GGCAGCGAAT GTACTGCTGA AAGTGATCAT CGAAACCGGC GAACTGAAAG ACGAAGCGCT
1521 GATCCGTAAA GCGTCTGAAA TCTCCATCAA AGCGGGTGCG GACTTCATCA AAACCTCTAC CGGTAAAGTG GCTGTGAACG
1601 CGACGCCGGA AAGCGCGCGC ATCATGATGG AAGTGATCCG TGATATGGGC GTAGAAAAAA CCGTTGGTTT CAAACCGGCG
1681 GGCGGCGTGC GTACTGCGGA AGATGCGCAG AAATATCTCG CCATTGCAGA TGAACTGTTC GGTGCTGACT GGGCAGATGC
1761 GCGTCACTAC CGCTTTGGCG CTTCCAGCCT GCTGGCAAGC CTGCTGAAAG CGCTGGGTCA CGGCGACGGT AAGAGCGCCA
1841 GCAGCTACGG ATCCTGACTC GAGCACCACC ACCACCACCA CTGAGATCCG GCTGCTAACA AAGCCCGAAA GGAAGCTGAG
            C-flank          His-Tag
1921 TTGGCTGCTG CCACCGCTGA GCAATAACTA GCATAACCCC TTGGGGCCTC TAAACGGGTC TTGAGGGGTT TTTTG
                                T7-Terminator
```

DeoC-EC-DM-NHalo AA-sequence *"NHalo"*

```
   1 MAEIGTGFPF DPHYVEVLGE RMHYVDVGPR DGTPVLFLHG NPTSSYVWRN IIPHVAPTHR CIAPDLIGMG KSDKPDLGYF
  81 FDDHVRFMDA FIEALGLEEV VLVIHDWGSA LGFHWAKRNP ERVKGIAFME FIRPIPTWDE WPEFARETFQ AFRTTDVGRK
 161 LIIDQNVFIE GTLPMGVVRP LTEVEMDHYR EPFLNPVDRE PLWRFPNELP IAGEPANIVA LVEEYMDWLH QSPVPKLLFW
 241 GTPGVLIPPA EAARLAKSLP NCKAVDIGPG LNLLQEDNPD LIGSEIARWL STLEISGLAE AAAKEAAAKE AAAKEAAAKM
 321 ASTDLKASSL RALKLMDLTT LNDDDTDEKV IALCHQAKTP VGNTAAIMIY PRFIPIARKT LKEQGTPEIR IATVTNFPHG
 401 NDDIDIALAE TRAAICYGAD EVDVVFPYRA LMAGNEQVGF DLVKACKEAC AAANVLLKVI IETGELKDEA LIRKASEISI
 481 KAGADFIKTS TGKVAVNATP ESARIMMEVI RDMGVEKTVG FKPAGGVRTA EDAQKYLAIA DELFGADWAD ARHYRFGASS
 561 LLASLLKALG HGDGKSASSY GS
```

pET21a-deoC-EC-DM-CHis_MCS-frame

```
GATC         Plasmid features
GATC         deoC-gene
GATC         gene modifications
GATC         HisTag

   1 AGATCTCGAT CCCGCGAAAT TAATACGACT CACTATAGGG GAATTGTGAG CGGATAACAA TTCCCCTCTA GAAATAATTT
                         T7-Promoter          lac-Operator
  81 TGTTTAACTT TAAGAAGGAG ATATACATAT GGCTAGCACT GATCTGAAAG CAAGCAGCCT GCGTGCACTG AAATTGATGG
                                     N-flank
 161 ACCTGACCAC CCTGAATGAC GACGACACCG ACGAGAAAGT GATCGCCCTG TGTCATCAGG CCAAAACTCC GGTCGGCAAT
 241 ACCGCCGCTA TCATGATCTA TCCTCGCTTT ATCCCGATTG CTCGCAAAAC TCTGAAAGAG CAGGGCACCC CGGAAATCCG
               C47M
 321 TATCGCTACG GTAACCAACT TCCCACACGG TAACGACGAC ATCGACATCG CGCTGGCAGA AACCCGTGCG GCAATCTGCT
                                                                                    A95C
 401 ACGGTGCTGA TGAAGTTGAC GTTGTGTTCC CGTACCGCGC GCTGATGGCG GGTAACGAGC AGGTTGGTTT TGACCTGGTG
 481 AAAGCCTGTA AAGAGGCTTG CGCGGCAGCG AATGTACTGC TGAAAGTGAT CATCGAAACC GGCGAACTGA AAGACGAAGC
 561 GCTGATCCGT AAAGCGTCTG AAATCTCCAT CAAAGCGGGT GCGGACTTCA TCAAAACCTC TACCGGTAAA GTGGCTGTGA
 641 ACGCGACGCC GGAAAGCGCG CGCATCATGA TGGAAGTGAT CCGTGATATG GGCGTAGAAA AAACCGTTGG TTTCAAACCG
 721 GCGGGCGGCG TGCGTACTGC GGAAGATGCG CAGAAATATC TCGCCATTGC AGATGAACTG TTCGGTGCTG ACTGGGCAGA
 801 TGCGCGTCAC TACCGCTTTG GCGCTTCCAG CCTGCTGGCA AGCCTGCTGA AAGCGCTGGG TCACGGCGAC GGTAAGAGCG
 881 CCAGCAGCTA CGGATCCCAT CACCATCACC ACCATTGACT CGAGCACCAC CACCACCACC ACTGAGATCC GGCTGCTAAC
              C-flank HisTag                            His-Tag
 961 AAAGCCCGAA AGGAAGCTGA GTTGGCTGCT GCCACCGCTG AGCAATAACT AGCATAACCC CTTGGGGCCT CTAAACGGGT
                                                           T7-Terminator
1041 CTTGAGGGGT TTTTTG
```

DeoC-EC-DM-CHis AA-sequence *"CHis"*

```
   1 MASTDLKASS LRALKLMDLT TLNDDDTDEK VIALCHQAKT PVGNTAAIMI YPRFIPIARK TLKEQGTPEI RIATVTNFPH
  81 GNDDIDIALA ETRAAICYGA DEVDVVFPYR ALMAGNEQVG FDLVKACKEA CAAANVLLKV IIETGELKDE ALIRKASEIS
 161 IKAGADFIKT STGKVAVNAT PESARIMMEV IRDMGVEKTV GFKPAGGVRT AEDAQKYLAI ADELFGADWA DARHYRFGAS
 241 SLLASLLKAL GHGDGKSASS YGSHHHHHH
```

pET21a-deoC-EC-DM-CHisNu_MCS-frame

```
GATC        Plasmid features
GATC        deoC-gene
GATC        gene modifications
GATC        HisNuTag

   1 AGATCTCGAT CCCGCGAAAT TAATACGACT CACTATAGGG GAATTGTGAG CGGATAACAA TTCCCCTCTA GAAATAATTT
                         T7-Promoter          lac-Operator
  81 TGTTTAACTT TAAGAAGGAG ATATACATAT GGCTAGCACT GATCTGAAAG CAAGCAGCCT GCGTGCACTG AAATTGATGG
                                      N-flank
 161 ACCTGACCAC CCTGAATGAC GACGACACCG ACGAGAAAGT GATCGCCCTG TGTCATCAGG CCAAAACTCC GGTCGGCAAT
 241 ACCGCCGCTA TCATGATCTA TCCTCGCTTT ATCCCGATTG CTCGCAAAAC TCTGAAAGAG CAGGGCACCC CGGAAATCCG
               C47M
 321 TATCGCTACG GTAACCAACT TCCCACACGG TAACGACGAC ATCGACATCG CGCTGGCAGA AACCCGTGCG GCAATCTGCT
                                                                                     A95C
 401 ACGGTGCTGA TGAAGTTGAC GTTGTGTTCC CGTACCGCGC GCTGATGGCG GGTAACGAGC AGGTTGGTTT TGACCTGGTG
 481 AAAGCCTGTA AAGAGGCTTG CGCGGCAGCG AATGTACTGC TGAAAGTGAT CATCGAAACC GGCGAACTGA AAGACGAAGC
 561 GCTGATCCGT AAAGCGTCTG AAATCTCCAT CAAAGCGGGT GCGGACTTCA TCAAAACCTC TACCGGTAAA GTGGCTGTGA
 641 ACGCGACGCC GGAAAGCGCG CGCATCATGA TGGAAGTGAT CCGTGATATG GGCGTAGAAA AAACCGTTGG TTTCAAACCG
 721 GCGGGCGGCG TGCGTACTGC GGAAGATGCG CAGAAATATC TCGCCATTGC AGATGAACTG TTCGGTGCTG ACTGGGCAGA
 801 TGCGCGTCAC TACCGCTTTG GCGCTTCCAG CCTGCTGGCA AGCCTGCTGA AAGCGCTGGG TCACGGCGAC GGTAAGAGCG
 881 CCAGCAGCTA CGGATCCCAT CACCATCACC ACCATGGCGG GTGTTGCTGA CTCGAGCACC ACCACCACCA CCACCACCAC
              C-flank HisNuTag                              His-Tag*
 961 TGAGATCCGG CTGCTAACAA AGCCCGAAAG GAAGCTGAGT TGGCTGCTGC CACCGCTGAG CAATAACTAG CATAACCCCT
                                                                          T7-Terminator
1041 TGGGGCCTCT AAACGGGTCT TGAGGGGTTT TTTG
```

* His8 instead of His6 due to misaligned primer. No consequence for gene.

DeoC-EC-DM-CHisNu AA-sequence “CHisNu”

```
   1 MASTDLKASS LRALKLMDLT TLNDDDTDEK VIALCHQAKT PVGNTAAIMI YPRFIPIARK TLKEQGTPEI RIATVTNFPH
  81 GNDDIDIALA ETRAAICYGA DEVDVVFPYR ALMAGNEQVG FDLVKACKEA CAAANVLLKV IIETGELKDE ALIRKASEIS
 161 IKAGADFIKT STGKVAVNAT PESARIMMEV IRDMGVEKTV GFKPAGGVRT AEDAQKYLAI ADELFGADWA DARHYRFGAS
 241 SLLASLLKAL GHGDGKSASS YGSHHHHHHG GCC
```

pET21a-deoC-EC-DM-CStrep_MCS-frame

```
GATC         Plasmid features
GATC         deoC-gene
GATC         gene modifications
GATC         StrepTag

   1 AGATCTCGAT CCCGCGAAAT TAATACGACT CACTATAGGG GAATTGTGAG CGGATAACAA TTCCCCTCTA GAAATAATTT
                         T7-Promoter          lac-Operator
  81 TGTTTAACTT TAAGAAGGAG ATATACATAT GGCTAGCACT GATCTGAAAG CAAGCAGCCT GCGTGCACTG AAATTGATGG
                                      N-flank
 161 ACCTGACCAC CCTGAATGAC GACGACACCG ACGAGAAAGT GATCGCCCTG TGTCATCAGG CCAAAACTCC GGTCGGCAAT
 241 ACCGCCGCTA TCATGATCTA TCCTCGCTTT ATCCCGATTG CTCGCAAAAC TCTGAAAGAG CAGGGCACCC CGGAAATCCG
               C47M
 321 TATCGCTACG GTAACCAACT TCCCACACGG TAACGACGAC ATCGACATCG CGCTGGCAGA AACCCGTGCG GCAATCTGCT
                                                                                      A95C
 401 ACGGTGCTGA TGAAGTTGAC GTTGTGTTCC CGTACCGCGC GCTGATGGCG GGTAACGAGC AGGTTGGTTT TGACCTGGTG
 481 AAAGCCTGTA AAGAGGCTTG CGCGGCAGCG AATGTACTGC TGAAAGTGAT CATCGAAACC GGCGAACTGA AAGACGAAGC
 561 GCTGATCCGT AAAGCGTCTG AAATCTCCAT CAAAGCGGGT GCGGACTTCA TCAAAACCTC TACCGGTAAA GTGGCTGTGA
 641 ACGCGACGCC GGAAAGCGCG CGCATCATGA TGGAAGTGAT CCGTGATATG GGCGTAGAAA AAACCGTTGG TTTCAAACCG
 721 GCGGGCGGCG TGCGTACTGC GGAAGATGCG CAGAAATATC TCGCCATTGC AGATGAACTG TTCGGTGCTG ACTGGGCAGA
 801 TGCGCGTCAC TACCGCTTTG GCGCTTCCAG CCTGCTGGCA AGCCTGCTGA AAGCGCTGGG TCACGGCGAC GGTAAGAGCG
 881 CCAGCAGCTA CGGATCCTCT GCGTGGTCAC ATCCTCAGTT CGAGAAATGA CTCGAGCACC ACCACCACCA CCACTGAGAT
              C-flank StrepTag                                  His-Tag
 961 CCGGCTGCTA ACAAAGCCCG AAAGGAAGCT GAGTTGGCTG CTGCCACCGC TGAGCAATAA CTAGCATAAC CCCTTGGGGC
                                                                        T7-Terminator
1041 CTCTAAACGG GTCTTGAGGG GTTTTTTG
```

DeoC-EC-DM-CStrep AA-sequence *“CStrep”*

```
   1 MASTDLKASS LRALKLMDLT TLNDDDTDEK VIALCHQAKT PVGNTAAIMI YPRFIPIARK TLKEQGTPEI RIATVTNFPH
  81 GNDDIDIALA ETRAAICYGA DEVDVVFPYR ALMAGNEQVG FDLVKACKEA CAAANVLLKV IIETGELKDE ALIRKASEIS
 161 IKAGADFIKT STGKVAVNAT PESARIMMEV IRDMGVEKTV GFKPAGGVRT AEDAQKYLAI ADELFGADWA DARHYRFGAS
 241 SLLASLLKAL GHGDGKSASS YGSSAWSHPQFEK
```

pET21a-deoC-EC-DM-CHalo_MCS-frame

```
GATC         Plasmid features
GATC         deoC-gene
GATC         gene modifications
GATC         linker-sequence
GATC         HaloTag

   1 AGATCTCGAT CCCGCGAAAT TAATACGACT CACTATAGGG GAATTGTGAG CGGATAACAA TTCCCCTCTA GAAATAATTT
                          T7-Promoter          lac-Operator
  81 TGTTTAACTT TAAGAAGGAG ATATACATAT GGCTAGCACT GATCTGAAAG CAAGCAGCCT GCGTGCACTG AAATTGATGG
                                       N-flank
 161 ACCTGACCAC CCTGAATGAC GACGACACCG ACGAGAAAGT GATCGCCCTG TGTCATCAGG CCAAAACTCC GGTCGGCAAT
 241 ACCGCCGCTA TCATGATCTA TCCTCGCTTT ATCCCGATTG CTCGCAAAAC TCTGAAAGAG CAGGGCACCC CGGAAATCCG
               C47M
 321 TATCGCTACG GTAACCAACT TCCCACACGG TAACGACGAC ATCGACATCG CGCTGGCAGA AACCCGTGCG GCAATCTGCT
                                                                                     A95C
 401 ACGGTGCTGA TGAAGTTGAC GTTGTGTTCC CGTACCGCGC GCTGATGGCG GGTAACGAGC AGGTTGGTTT TGACCTGGTG
 481 AAAGCCTGTA AAGAGGCTTG CGCGGCAGCG AATGTACTGC TGAAAGTGAT CATCGAAACC GGCGAACTGA AAGACGAAGC
 561 GCTGATCCGT AAAGCGTCTG AAATCTCCAT CAAAGCGGGT GCGGACTTCA TCAAAACCTC TACCGGTAAA GTGGCTGTGA
 641 ACGCGACGCC GGAAAGCGCG CGCATCATGA TGGAAGTGAT CCGTGATATG GGCGTAGAAA AAACCGTTGG TTTCAAACCG
 721 GCGGGCGGCG TGCGTACTGC GGAAGATGCG CAGAAATATC TCGCCATTGC AGATGAACTG TTCGGTGCTG ACTGGGCAGA
 801 TGCGCGTCAC TACCGCTTTG GCGCTTCCAG CCTGCTGGCA AGCCTGCTGA AAGCGCTGGG TCACGGCGAC GGTAAGAGCG
 881 CCAGCAGCTA CGGATCCGAA GCAGCGGCCA AAGAAGCTGC GGCCAAAGAG GCAGCCGCGA AAGAAGCAGC GGCGAAAGCA
              C-flank 4x EAAAK-linker
 961 GAAATTGGTA CGGGATTTCC GTTTGACCCG CATTATGTGG AGGTTCTGGG TGAACGCATG CACTACGTGG ATGTTGGTCC
1041 GCGCGATGGC ACACCGGTGC TGTTTCTGCA TGGTAATCCG ACCTCCAGCT ATGTTTGGCG CAACATTATT CCGCATGTCG
1121 CCCCAACGCA TCGCTGTATT GCCCCAGATC TCATTGGCAT GGGCAAAAGC GACAAACCGG ATTTGGGCTA CTTCTTCGAC
1201 GATCACGTAC GGTTTATGGA CGCCTTTATC GAGGCTCTGG GACTGGAGGA AGTAGTGCTG GTTATTCATG ACTGGGGCTC
                                                 C-->G (silent)
1281 TGCATTAGGC TTTCACTGGG CTAAACGGAA CCCAGAACGC GTCAAGGGGA TTGCCTTCAT GGAGTTCATC CGTCCGATTC
1361 CGACCTGGGA TGAATGGCCC GAATTTGCCC GTGAAACCTT TCAGGCGTTT CGTACCACGG ATGTTGGCCG TAAGCTCATC
1441 ATCGACCAAA ACGTGTTCAT TGAGGGCACT CTTCCCATGG GAGTAGTGCG TCCTTTAACC GAAGTCGAGA TGGACCACTA
1521 TCGCGAACCC TTCCTGAATC CGGTTGATCG CGAACCGCTG TGGCGCTTCC CGAATGAGCT GCCTATTGCT GGTGAACCGG
1601 CGAATATCGT GGCACTTGTG GAAGAATACA TGGATTGGCT GCATCAGAGT CCAGTCCCTA AGCTGTTGTT TTGGGGTACA
1681 CCTGGCGTGT TGATTCCGCC TGCAGAAGCT GCTCGCTTAG CGAAAAGCTT GCCCAACTGC AAAGCGGTCG ATATTGGGCC
1761 AGGTCTGAAC CTGTTACAGG AGGATAACCC GGATCTGATC GGGAGTGAAA TCGCGCGTTG GCTGTCAACT CTGGAAATCT
1841 CGGGTCTTGC ATCCTGACTC GAGCACCACC ACCACCACCA CTGAGATCCG GCTGCTAACA AAGCCCGAAA GGAAGCTGAG
                            His-Tag
1921 TTGGCTGCTG CCACCGCTGA GCAATAACTA GCATAACCCC TTGGGGCCTC TAAACGGGTC TTGAGGGGTT TTTTG
                                   T7-Terminator
```

DeoC-EC-DM-CHalo AA-sequence *“CHalo”*

```
  1 MASTDLKASS LRALKLMDLT TLNDDDTDEK VIALCHQAKT PVGNTAAIMI YPRFIPIARK TLKEQGTPEI RIATVTNFPH
 81 GNDDIDIALA ETRAAICYGA DEVDVVFPYR ALMAGNEQVG FDLVKACKEA CAAANVLLKV IIETGELKDE ALIRKASEIS
161 IKAGADFIKT STGKVAVNAT PESARIMMEV IRDMGVEKTV GFKPAGGVRT AEDAQKYLAI ADELFGADWA DARHYRFGAS
241 SLLASLLKAL GHGDGKSASS YGSEAAAKEA AAKEAAAKEA AAKAEIGTGF PFDPHYVEVL GERMHYVDVG PRDGTPVLFL
321 HGNPTSSYVW RNIIPHVAPT HRCIAPDLIG MGKSDKPDLG YFFDDHVRFM DAFIEALGLE EVVLVIHDWG SALGFHWAKR
401 NPERVKGIAF MEFIRPIPTW DEWPEFARET FQAFRTTDVG RKLIIDQNVF IEGTLPMGVV RPLTEVEMDH YREPFLNPVD
481 REPLWRFPNE LPIAGEPANI VALVEEYMDW LHQSPVPKLL FWGTPGVLIP PAEAARLAKS LPNCKAVDIG PGLNLLQEDN
561 PDLIGSEIAR WLSTLEISGL AS
```

Supplementary References

1. Schägger, H.; von Jagow, G. Tricine-sodium dodecyl sulfate-polyacrylamide gel electrophoresis for the separation of proteins in the range from 1 to 100 kDa. *Anal. Biochem.* **1987**, *166*, 368-379, doi:https://doi.org/10.1016/0003-2697(87)90587-2.
2. Kang, D.; Gho, Y.S.; Suh, M.-K.; Kang, C. Highly Sensitive and Fast Protein Detection with Coomassie Brilliant Blue in Sodium Dodecyl Sulfate-Polyacrylamide Gel Electrophoresis. *Bull. Korean Chem. Soc.* **2002**, *23*, 1511-1512, doi:10.5012/BKCS.2002.23.11.1511.
3. Details of R square. Available online: https://www.originlab.com/doc/en/Origin-Help/Details_of_R_square (accessed on 14.07.2022).
4. Entry P0A6L0 · DEOC_ECOLI. Available online: https://www.uniprot.org/uniprotkb/P0A6L0 (accessed on 20.07.2022).
